# The Seeking Proxies for Internal States (SPIS) Model of OCD – A Comprehensive Review of Current Findings and Implications for Future Directions

**DOI:** 10.2174/1570159X21666230920165403

**Published:** 2023-09-21

**Authors:** Amit Lazarov, Nira Liberman, Reuven Dar

**Affiliations:** 1 School of Psychological Sciences, Tel Aviv University, Tel Aviv 69978, Israel

**Keywords:** Obsessive-compulsive disorder, obsessions, compulsions, internal states, proxies, doubt

## Abstract

The Seeking Proxies for Internal States (SPIS) model of obsessive-compulsive disorder (OCD) explains symptoms of OCD as stemming from attenuated access to internal states, which is compensated for by using proxies, which are indices of these states that are more discernible or less ambiguous. Internal states in the SPIS model are subjective states that are not accessible to others, encompassing physiological states, motivations, preferences, memories, and emotions. Compensatory proxies in OCD include fixed rules and rituals as well as seeking and relying on external information. In the present review, we outline the SPIS model and describe its basic tenets. We then use the SPIS conceptualization to explain two pivotal OCD-related phenomena - obsessive doubt and compulsive rituals. Next, we provide a detailed overview of current empirical evidence supporting the SPIS in several domains, including physiological states, emotions, sense of understanding, decision-making, and sense of agency. We conclude by discussing possible neural correlates of the difficulty in accessing internal states, focusing on the anterior insular cortex (AIC) and highlighting potential clinical implications of the model to the treatment of OCD.

## 1 INTRODUCTION

Obsessive-compulsive disorder (OCD) is defined by the occurrence of obsessions, which are recurrent and persistent thoughts, urges, images, or impulses experienced as intrusive and unwanted, and typically cause marked anxiety or distress; and compulsions, which are repetitive behaviors (*e.g*., hand washing, ordering, checking) or mental acts (*e.g*., praying, counting, repeating words silently) the individual feels the urge to perform in response to an obsession and/or according to fixed and rigid rules [1]. Echoing this phenomenological definition of the disorder, most extant OCD models postulate that compulsive behavior is performed primarily to reduce anxiety and negative emotions brought on by obsessions ([2]; for reviews, see [3, 4]).

Recently, we proposed a model of OCD called the Seeking Proxies for Internal States (SPIS) model [5-8]. This model diverges from extant OCD models in assuming that compulsive behaviors have a functional role beyond mere anxiety reduction; namely, they serve as indicators or substitutes for vague or ambiguous internal states. The SPIS model proposes that obsessions are associated with attenuated accesses to one’s own internal states (*e.g*., feelings, preferences, physiological states), a condition that drives the individual to seek and rely on proxies – compensatory substitutes which are perceived by the individual as more discernible and/or less ambiguous than the internal states for which they substitute. These compensatory proxies typically comprise fixed and rigid rules, rituals, or reliance on external information.

This comprehensive review describes the SPIS model, elaborating on its two core tenets - attenuated access to internal states and reliance on proxies. We then describe the model’s conceptualization of two main OCD-related phenomena – compulsive behavior and obsessional doubt and discuss the relation of the SPIS model to other prominent theories of OCD. Next, we present some empirical support for the SPIS model and conclude by speculating on the potential underlying neural correlates of attenuated access to internal states, focusing specifically on the anterior insular cortex (AIC) and highlighting possible clinical implications of the model.

## 2 CORE COMPONENTS OF THE SEEKING PROXIES FOR INTERNAL STATES (SPIS) MODEL OF OCD

### 2.1 Attenuated Access to Internal States

The first core assertion of the SPIS model is that OCD is characterized by diminished access to internal states – subjective states that are not, and cannot, be directly shared by others. Internal states can be affective/evaluative (*e.g*., emotions, preferences), cognitive (*e.g*., perception, memory, comprehension), or physical (*e.g*., relaxation, interoception). According to the SPIS model, obsessive-compulsive (OC) individuals^1^ find it difficult to know, for example, what they feel, what they prefer, whether they understand something correctly, or how tense their muscles are. When faced with a question about their internal states, provided that the answer is important for them (*e.g*., do I feel satisfied with how much I secured my house), this difficulty will come into play, leaving them in doubt, anxiously searching for alternative sources of information, which we term proxies [5, 9]^2^.

### 2.2 Proxies (For Internal States)

The second core component of the SPIS model is proxies, which are indices of the internal state that a person with OCD perceives as more discernible and/or less ambiguous than the internal states that they index. Proxies may comprise rules, procedures, behaviors or environmental stimuli [5]. In the SPIS model, OC individuals seek, use, and rely on such proxies in an attempt to compensate for their attenuated access to their internal states. To use a common clinical manifestation of OCD, that of contamination and cleaning, a hand-washing ritual can serve as an objective proxy that signals to the person with OCD that they have washed their hands sufficiently, circumventing the need to access an internal sense of cleanliness or one of satisfaction with the results of washing^3^. A second example from the clinical realm of relationship-focused OCD (ROCD; [10, 11]), in which people monitor and question their own feelings towards their intimate partner, would be an individual who resorts to monitoring how often to text their partner, or how much they spend on buying them gifts, to indicate how much love they feel for their partner (*i.e*., the internal state).

As these two clinical examples illustrate, the notion of proxies in the SPIS mode implies that OCD rules and rituals are functional – they substitute and thereby compensate for deficient access to one's own internal states. Similarly, a fixed ritual of checking that the stove is off (*e.g*., turning the gas knob on and off a fixed number of times and in a fixed pattern) might compensate for a more evasive feeling that one has checked enough and that it is safe to leave the kitchen [12]. The central proposition of the SPIS model that OCD rules and rituals compensate for difficult-to-access internal states, is inspired by the classic account of David Shapiro [13]. According to Shapiro, OC individuals find it difficult to know their own feelings, wishes and preferences directly, which leaves them in a constant experience of uncertainty and self-doubt. According to Shapiro, and in line with the SPIS model, this deficit forces them to rely on external indicators to infer these internal states. Shapiro likened people with “obsessive-compulsive style” to pilots who navigate at night and must rely on flight instruments instead of on their own eye sights. According to Shapiro, such individuals have lost the natural sense of “conviction” and must rely on rules or norms to answer questions regarding their own feelings and motivations. As concluded by Shapiro, “nothing in (the OC person’s) situation is experienced directly; only indicators are experienced, things that indicate other things [13]”.

Importantly, relying on proxies for internal states has significant drawbacks. First, proxies may vary in their validity. While some proxies may be valid indices of specific internal states (*e.g*., free time spent solving math quizzes as an indicator of liking math), others may be weak indicators of the relevant internal states (*e.g*., knowing a text by heart is a dubious indicator of comprehension). Regardless, focusing attention and effort on a weak proxy may end up leading one away from the relevant internal state (*e.g*., focusing on learning study material by heart may actually disrupt the effort to understand it). Moreover, proxies often lose their apparent clarity at close examination, leading to further substitution. An example might help to illustrate this process. A woman with OCD was uncertain about her level of happiness with her partner, and to gauge her happiness, she resorted to analyzing her facial expressions in 'selfies' taken with him. However, this proxy did not provide a sufficiently clear answer, leading her to search for more “objective” indicators that were ever more distant from the elusive feeling of love. One such indicator was the number of text messages she sent him each day. Another problem is that some proxies may appear effective in the short term but fail to fulfill their purpose in the long run. For example, a man with OCD was fearful of the possibility that he was attracted to men. He adopted a habit of watching gay adult films and assessing his level of sexual arousal. However, this kind of proxy is inherently short-lived, as not experiencing arousal on a specific occasion does not guarantee a similar outcome in the future. Consequently, such proxies are bound to be ineffective in providing the desired information regarding one's internal state, leaving individuals with OCD trapped in an endless cycle of self-testing and uncertainty. We will elaborate on this point in more detail later when we discuss how the SPIS model explains obsessive doubt.

## 3 THE SPIS MODEL ACCOUNT OF OBSESSIVE DOUBT AND COMPULSIVE RITUALS

### 3.1 Obsessive Doubt

Among the most prominent characteristics of OCD is obsessive doubt, which can trigger a variety of symptoms such as excessive checking, compulsive washing, mental reconstruction efforts, self-monitoring, and never-ending efforts to obtain validation and reassurance from others [14]. The role of endemic doubt in the etiology and phenomenology of OCD has been discussed by classical [13, 15-17] and modern theoreticians [12, 18], generally defined as a “lack of subjective certainty about and confidence in one’s perceptions and internal states” [19]. Research-wise, obsessional doubts have been demonstrated in relation to a variety of domains, including, among others, memory and perception (for a review and meta-analysis, see [20]), decision-making and concentration [21, 22], task-completion [23, 24], the self-concept [25, 26], and intimate relationships [11].

The SPIS model assigns to doubt a central role in the phenomenology and etiology of OCD, similar to the previous models of the disorder. However, elaborating these earlier models, the SPIS model also provides an account of the repetitive nature of obsessional doubt. Specifically, the SPIS model characterizes obsessional doubt as the cycle that follows the stages depicted in Fig. (**1**). Attempting to access the pertinent internal state, encountering a lack of clarity or a definitive answer, and then attempting to access the internal states again or searching for a substitute proxy, all of which can perpetuate further looping through these stages. Hence, obsessional doubt, as conceptualized by the SPIS model, arises from two (necessary) conditions: A strong wish to access an internal state (a “question”) and diminished access to that state, a combination that sets in motion the potentially repetitive process depicted in Fig. (**1**)^4^.

The SPIS model, therefore, postulates two separate theoretical constructs: the challenge of accessing one's internal states and the subsequent obsessional doubt. It is important to note that difficulty in accessing internal states is a necessary requirement but not the sole determinant of obsessional doubt. Additionally, the causal relationship between the two is not unidirectional. On the one hand, the lack of clarity regarding internal states can lead to doubt; on the other, the presence of obsessional doubt, which involves cycling through the steps shown in Fig. (**1**), can undermine the clarity of the internal state that an individual is attempting to access. This bi-directional relationship between doubt and attenuation of internal states may eventually give rise to a vicious cycle: doubt can reduce access to internal states, and impaired access to internal states, in turn, is likely to further increase doubt regarding these states.

While the logic according to which diminished access to internal states may lead to obsessive doubt is fairly intuitive, accounting for the reverse effect (*i.e*., from obsessive doubt to attenuation of internal states) is less straightforward. One possibility would be that obsessive doubt increases efforts to identify, experience and monitor the relevant internal state, which only serves to further degrade that very experience [27]. For example, repeatedly questioning whether I am enjoying my coffee would likely dampen any sense of enjoyment that might have been there initially. Likewise, doubting one's feelings towards one's partner would elicit monitoring and repeated examination of these feelings, which might paradoxically diminish one’s ability to feel them. This notion resonates with the well-established finding that repeated checking (*e.g*., [28, 29]) and even prolonged staring (*e.g*., [30, 31]) lead to diminished quality of one’s memory and perception. Also, as we describe in detail below, our own experimental evidence suggests that undermining individuals’ confidence in their ability to accurately assess their internal states impairs performance on tasks that require access to these internal states [32-34].

### 3.2 Compulsive Behavior

In the Diagnostic and Statistical Manual of Mental Disorders criteria (DSM-5; [1]), compulsive rituals are defined as “repetitive behaviors or mental acts that the individual feels driven to perform in response to an obsession or according to rules that must be applied rigidly. These behaviors or mental acts are aimed at preventing or reducing anxiety or distress, or preventing some dreaded event or situation; however, they are not connected in a realistic way with what they are designed to neutralize or prevent, or are clearly excessive.” Two facets of this definition are essential for understanding compulsive rituals within the SPIS model: First, compulsions are goal-directed; second, compulsions are governed by rules that seem dissociated from those goals and can be best understood as proxies. Such proxies exempt the OC individual from relying on evasive internal states, like the sense of cleanliness or satisfaction with one’s actions. Importantly, many explicit goals of compulsions (*e.g*., avoiding disease by cleaning one’s hands) typically have no clear end-state. When one pursues goals with no clear end-states, such as escaping danger, cleaning the house, or painting a beautiful picture, the feeling of having done enough often serves as a stopping rule [35, 36]. Hence, an OC person, who finds it difficult to access their feeling of satisfaction (*i.e*., the feeling of having done enough), would seek other criteria for stopping. Rituals can provide such stopping criteria, and according to the SPIS model, this is their primary function.

The SPIS conceptualization of compulsive behaviors as proxies for vague internal states echoes other theories of OCD that view compulsive behavior as resulting from a failure of feedback systems to generate clear signals of goal attainment. These signals include the sense of completion [23, 24], safety [12, 18], certainty [37, 38] or a “just right” feeling [39-41]. For example, according to Szechtman and Woody [12], OCD is related to a disturbance in the “feeling of knowing,” an internally generated feeling serving as a termination signal for a security motivation system, which is responsible for initiating security-related behaviors in response to specific threats in the environment. In individuals with OCD, the system’s behavioral output (*i.e*., the security-related behaviors) fails to generate this feeling, leaving the security motivation system in a continuously operating mode. Failing to generate the signal that would normally end the operation of the system, the security-related behavior is repeated, thus manifesting as a compulsive behavior. Boyer and Lienard [18] similarly suggest that OCD rituals result from a failure of one's actions to trigger “satiety feedback feelings” that normally terminate the operation of an evolutionary “precaution system.” And according to “just right” models [39-41], OCD is characterized by a failure to achieve a “just right” feeling, leading a person with OCD to engage in repetitive, compulsive behaviors.

The SPIS model shares important features with these theoretical accounts but diverts from them in two important respects. First, some of these models are limited to specific domains or OCD-related contents, such as safety and security [12, 18] or incompleteness [23, 24]. Conversely, the SPIS model is not bound to specific content domains. As we will demonstrate later, some of the findings supporting the SPIS model involve internal states that have little to do with typical OC concerns [6, 32, 33, 42]. The only necessary conditions for setting in motion the process described by the SPIS model are that a person wishes to answer a query regarding *any* internal state and that their access to that internal state is attenuated. Second, in the above-mentioned models, compulsions are seen as non-functional byproducts of a malfunctioning goal-directed system. According to the DSM-5 definition, however, compulsive rituals are not just repetitive actions. They are governed by strict rules regarding how they are performed or when they should end, and they often provide relief or alleviate distress. The SPIS model further addresses the functional role of compulsions, proposing that they serve as proxies that substitute for ambiguous internal states, effectively signaling progress towards or completion of a goal [27].

## 4 EMPIRICAL SUPPORT FOR THE SPIS MODEL

In this section, we describe various studies that examined two main predictions of the SPIS model: (1) compared to non-OC participants, OC participants would show more performance deficits on tasks that require accurate access to their own internal states^5^; and (2) compared to non-OC participants, OC participants would rely more on proxies (both valid and non-valid) when asked to assess their internal states.

### 4.1 Bodily Internal States

In this set of studies, bodily states were used as internal states, and a biofeedback apparatus served as an available proxy. Two experimental paradigms were used – a genuine feedback paradigm, examining participants’ ability to achieve an internal state, and a false feedback paradigm gauging participants’ ability to accurately assess their own bodily states. Two bodily states were examined in each paradigm – relaxation, indexed by Galvanic skin responses (GSR), and muscle tone, indexed by electromyography (EMG).

#### 4.1.1 Genuine Feedback Paradigm

Participants were asked to achieve a bodily internal state (*i.e*., relaxation or muscle tension), and their accuracy in attaining the required internal state was assessed. In the relaxation version of this paradigm, participants were asked to try to relax, while in the muscle tension version, they were required to produce (four) different levels of muscle tone after being trained on two anchor levels^6^. In Phase 1 of this paradigm, participants had to achieve the internal state while not being able to view/use the biofeedback monitor. Prior to Phase 2, participants were given a short explanation as to the nature and function of the biofeedback apparatus, including a 2-minute period during which they could test it and familiarize themselves with it. Phase 2 was identical to Phase 1, except that participants could see the biofeedback monitor^7^. Phase 3 replicated Phase 1 (*i.e*., the biofeedback monitor was not available) – participants had to achieve the internal state while not being able to view/use the biofeedback monitor. Prior to Phase 4, participants were informed that they would not be able to view the biofeedback monitor continuously during the next phase but that they will be offered several opportunities to do so. They were warned, however, that choosing to view the monitor might disrupt their concentration and hence impair their performance on the task.

##### 4.1.1.1 Analog Samples^8^

**Fig. (2):**
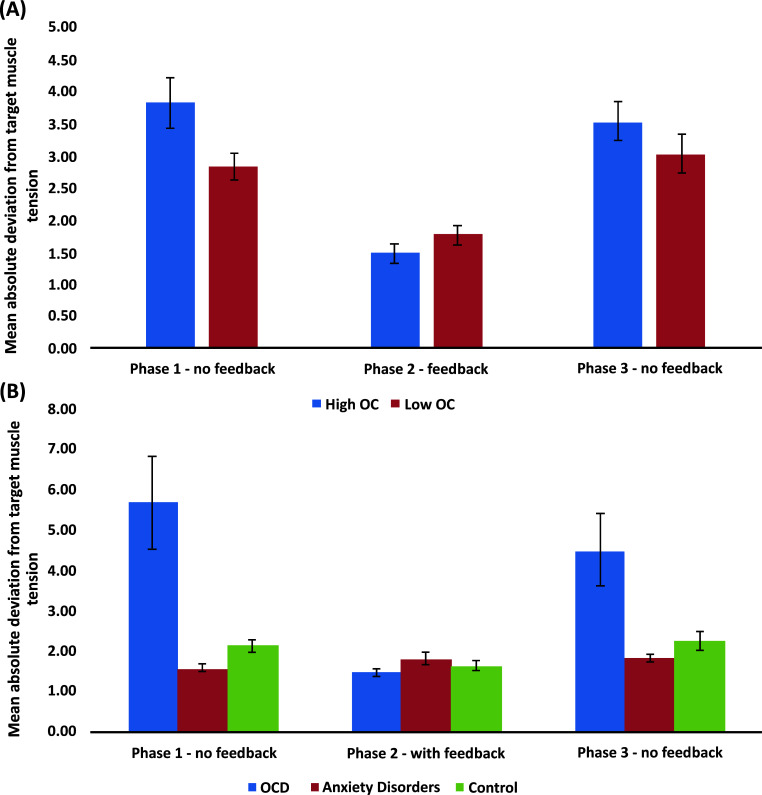
Mean absolute deviations from target muscle tension by Phase and Group for (**A**) analog samples comprised of participants with high and low levels of obsessive-compulsive (OC) symptoms; and (**B**) a clinical sample of OCD patients, anxiety disorders patients, and non-clinical control participants.

Comparing participants with high and low levels of OC symptoms (Fig. **2A**) showed similar findings across the two versions of this paradigm (relaxation: [43], Study 1; muscle tension: [42]): High OC participants were less accurate than low OC participants in the absence of biofeedback, with no group differences emerging when the biofeedback was available. High OC participants also requested the biofeedback monitor more times than low OC participants in Phase 4, despite the potential cost in performance they had been cautioned about. Importantly, in both studies, the deficient performance of high OC participants was evident from actual physiological measures – GSR for relaxation and EMG for muscle tone – rather than mere self-reports. Thus, performance-related self-doubt or reduced confidence alone cannot explain these results.

##### 4.1.1.2 Clinical Samples

To extend our findings to clinical OCD and tease apart the potential effects of OCD from those of anxiety and depression, which commonly co-occur with OCD [44], we compared the performance of OCD participants, matched anxiety disorder participants, and non-clinical controls on the muscle tensing version of the paradigm [6]^9^. Results fully replicated the findings with analog samples [42]: (1) in the absence of biofeedback, OCD participants were less accurate in their muscle tension production than both anxious participants and non-clinical controls; (2) in the presence of biofeedback, no group differences emerged; and (3) OCD participants requested to see the biofeedback monitor significantly more times than both anxiety disorder and non-clinical participants, despite being aware of the potential cost in performance. Importantly, as can be seen in Fig. (**2B**), group differences were much more prominent than those previously obtained with the analog samples (Fig. **2A**), strongly supporting the generalizability of our findings to clinical OCD. Moreover, as the OCD and anxiety groups had similar levels of anxiety and depression, these findings further corroborated the specificity of our findings to OCD rather than to these other disorders.

##### 4.1.1.3 Manipulating Confidence

Here we were interested in exploring whether inducing doubt and increasing monitoring of one’s internal states would yield a result pattern similar to that observed among OC participants and OCD patients. To that end, we implemented a “confidence undermining” manipulation in the muscle tone procedure described above among non-selected participants. Specifically, half of the participants were told prior to the muscle tone production task that “people often feel too confident about their ability to control their muscle tension, so [they] should monitor themselves closely and repeatedly to make sure that [they] are correctly and accurately producing the required muscle tension levels” [32]. As predicted, the results closely mimicked the ones obtained with the analog and clinical OCD sample: In the absence of the biofeedback monitor (Phase 1), the manipulation group was less accurate in producing the different muscle tension levels, and in Phase 4, requested the monitor more times than the control group, which did not receive the confidence undermining instructions. It is important to emphasize that the manipulation impaired the accuracy of actual behavioral performance rather than only self-reported confidence. This finding indicates that the manipulation not only made participants less confident (as reflected in their requests to receive feedback in Phase 4) but also made them less able to correctly reproduce the designated levels of muscle tension (Phase 1). In the terminology of the SPIS model, the manipulation that induced doubt and monitoring of internal states also reduced participants’ ability to accurately achieve the required internal state.

#### 4.1.2 False Feedback Paradigm

Self-perception theory suggests that people learn about or infer their attitudes and preferences by observing their own overt behavior and that this is especially likely to occur when “*internal cues are weak, ambiguous, or uninterpretable*” ([45], p. 2). According to the SPIS model, this condition is typical for OC individuals, which implies that self-perception-based inferences might be particularly prominent in OCD. We employed a commonly used method to assess self-perception effects; namely, we gauged the extent to which participants are influenced by false feedback when judging their own attitudes, preferences, or emotions ([46]; for reviews, see also [47, 48]).

In this series of studies, participants were asked to assess their internal state (relaxation or muscle tone) after receiving relevant but false physiological feedback. In the relaxation version of this paradigm, participants were once again asked to try to relax deeply, while in the muscle tension version, they were asked to “let go” of any tension in their forearm muscles. The false feedback paradigm involved two phases that were counterbalanced. In each phase, participants were presented with pre-programmed “biofeedback” that displayed their physiological state. In one phase, the biofeedback monitor showed a descending line graph, indicating a decrease in the relevant physiological state, while in the other phase, an ascending line graph indicated an increase in the same state. After each phase, participants were asked to assess their own physiological state by rating how anxious (*versus* relaxed) they felt or evaluating the tension (*versus* looseness) in their forearm muscles. We examined the impact of the false feedback on participants' estimations of their internal states while simultaneously also recording participants’ actual physiological state. Based on the SPIS model and the predictions of self-perception theory, we predicted the OC participants, compared to non-OC participants, would show stronger reliance on the provided false feedback when assessing their internal states of relaxation and muscle tension.

##### 4.1.2.1 Analog Samples

As predicted, high OC participants were more affected than low OC participants by the false feedback in evaluating the relevant internal states – either relaxation ([43], Study 2) or muscle tone ([33], Study 1; see [34] for a replication). Importantly, the false feedback did not affect the actual physiological measures (GSR levels in the relaxation study; EMG levels in the muscle tone one), but only the subjective evaluations (presumably, self-perception-based inferences) of these states.

##### 4.1.2.2 Clinical Samples

We conducted the above-described muscle tension study [33] among OCD participants, matched anxiety disorder participants, and non-clinical controls. Echoing our results with the analog samples, OCD participants were more affected by false biofeedback when judging their own muscle tension, as compared to both anxiety disorder and non-clinical control participants [6]. These findings demonstrated, once again, that the predictions of the SPIS model not only generalize to clinical OCD but are also *specific* to OCD – participants with anxiety disorder did not differ from non-clinical control participants – both groups showed no effect of the false feedback on their evaluations of their own muscle tension. Importantly, here, too, the groups did not differ when examining actual physiological (EMG) levels.

##### 4.1.2.3 Manipulating Confidence

Using a similar experimental approach to that taken in the genuine feedback paradigm, we examined whether experimentally inducing doubt in and increasing monitoring of one’s ability to accurately assess their internal states would increase reliance on the false biofeedback. As in [32], we told half of the participants in the relaxation version of the false feedback procedure that people often feel too confident about their ability to relax; hence they should monitor themselves closely and repeatedly to make sure that they are indeed relaxed. As predicted and in line with our previous results, the manipulation increased the effects of the false feedback on perceived levels of relaxation, with no group differences in the actual physiological measure ([33]; see [34] for replication with muscle tension).

### 4.2 Emotional States

The next series of studies examined the SPIS tenets in the realm of emotions, exploring participants' access to their own affective states, using two experimental approaches – an emotional intelligence (EI) paradigm and an emotional reaction assessment paradigm.

#### 4.2.1 The Emotional Intelligence (EI) Paradigm

In the first paradigm, we used a performance-based test of emotional intelligence (EI), namely, the Mayer Salovey Caruso Emotional Intelligence Test (MSCEIT; [49-51]), to examine access to internal emotional states. The MSCEIT assesses performance in two areas of EI, Experiential and Strategic. The clear distinction between these two areas makes the MSCEIT ideal for testing the SPIS model in the realms of emotions. Experiential Emotional Intelligence (EI) reflects the ability to perceive accurately one's emotional states, which relies on directly accessing one's affective state. Conversely, Strategic EI involves the cognitive understanding and management of emotions and relies on a semantic understanding of emotions rather than on direct experience^10^.

In the studies described in this section, participants were tested individually or in groups of up to four individuals in a small and quiet room. Upon arrival, they received a brief explanation of the experiment and provided informed consent and then completed the MSCEIT. According to the SPIS model, individuals with OCD are expected to perform poorly on Experiential EI items, as these require accurately accessing one's own emotional states. However, their performance on Strategic EI items, which rely on semantic knowledge and normative rules about expected emotional responses in certain situations, should not be impaired (see [52] for a similar distinction between affective and semantic processes in emotion).

##### 4.2.1.1 Analog Samples

As depicted in Fig. (**3A**), comparing participants with high and low levels of OC symptoms showed that, as predicted by the SPIS model, high OC participants had lower scores than low OC participants on the Experiential but not on the Strategic part of the MSCEIT ([9], Study 1). As the accurate perception of one’s own emotional states is necessary for performing adequately only on Experiential EI items but not on Strategic EI items, this pattern of results is consistent with a deficiency in perceiving and experiencing affective states. Given that low scores on the MSCEIT reflect wrong answers (relative to established norm ratings), this poorer performance does not merely reflect low confidence in one’s performance but rather poor performance on items that require one to access emotional states.

In another study ([9], Study 2), we examined the relationship between OC symptoms and the two EI areas across the full range of OC scores, also exploring the specificity of this relationship to OC symptoms over depression and anxiety symptoms. A sample of unselected participants completed the MSCEIT and measures of OCD symptoms (the OCI-R; [53]), depression and anxiety (the Depression, Anxiety and Stress Scales-21 (DASS-21; [54], respectively). As predicted, OCI-R scores were negatively correlated with Experiential EI but not with Strategic EI, and the correlation remained statistically significant even after controlling for symptoms of both anxiety and depression.

##### 4.2.1.2 Clinical Samples

**Fig. (3):**
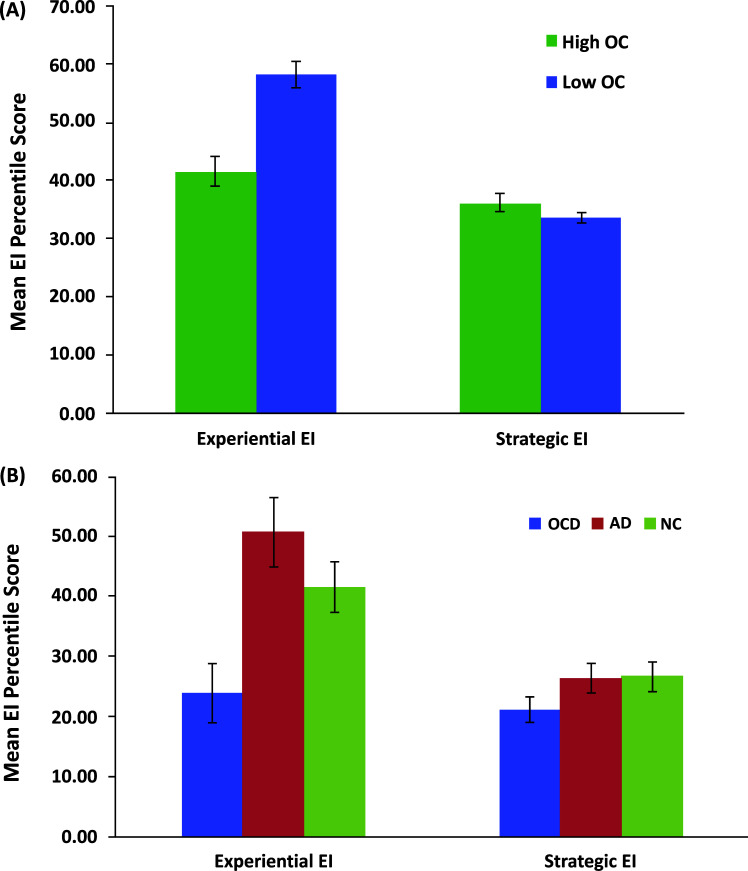
Mean Emotional Intelligence (EI) percentile score by EI area (Experiential, Strategic) and Group for (**A**) analog samples comprised of participants with high and low levels of obsessive-compulsive (OC) symptoms; and (**B**) a clinical sample of OCD patients, anxiety disorders (AD) patients, and non-clinical (NC) control participants.

Extending these findings to clinical OCD, we tested participants with OCD, control participants with anxiety disorders (AD) and non-clinical (NC) control participants (matched on age, gender, and years of education) on the MSCEIT [7]. Replicating our previous findings with the analog samples, there was a significant group difference in the Experiential area but not in the Strategic area of the MSCEIT. Specifically, participants with OCD had lower scores than AD and NC participants, with no significant differences between the two control groups (Fig. **3B**). These results not only extended our findings to clinical OCD, but also demonstrated their specificity to OCD over anxiety and/or depression.

##### 4.2.1.3 Manipulating Confidence

Dar and colleagues [9] gave half of a non-selected participant pool instructions that undermined their confidence in their ability to accurately assess their own emotions. As predicted, compared with control participants, participants in the experimental condition had lower scores on the Experiential but not on the Strategic area of the MSCEIT, echoing our previous findings described above.

#### 4.2.2 Emotional Reaction Assessment Paradigm

In the next set of studies, participants were asked to assess and rate their emotional reactions to well-validated emotional pictures (*i.e*., pictures with established norm ratings). We predicted that OC symptoms would be associated with less accuracy and operationalized as more divergent from the norm ratings.

##### 4.2.2.1 Analog Samples

In the first study ([55], Study 1), high and low OC participants rated how they felt towards photos with positive, neutral, and negative valence that were taken from the International Affective Picture System [56] (pictures were matched on arousal levels). Mean deviation from average norm ratings (of the IAPS picture system) of each valence type (positive, neutral, negative) served as the primary outcome measure. As predicted, high OC participants’ mean deviation score was significantly higher (*i.e*., they deviated more from norm ratings), compared with low OC participants, across all three valence groups (*i.e*., for positive, neutral and negative pictures). High OC participants also rated the task as more difficult than low OC participants.

The second study ([55], Study 2) employed a similar procedure (*i.e*., participants were asked to rate how they felt while viewing positive, neutral, and negative pictures) but used a more recently developed picture dataset (*i.e*., the Basic-Emotions Nencki Affective Picture System (NAPS-BE; [57]) in a sample of non-selected participants. This study aimed to investigate the relationship between obsessive-compulsive disorder (OC) symptoms, affective ratings, and symptoms of depression and anxiety across a wide range of OCD symptom scores. The findings revealed that only OCD symptoms showed a positive correlation with mean deviation scores, while symptoms of depression and anxiety did not demonstrate the same correlation. We also examined the difference between high and low OC participants, using the OCI-R cutoff score, which yielded similar results to those observed in Study 1 mentioned earlier. Overall, the results from both studies indicate that individuals with high levels of OCD symptoms have difficulty accurately assessing their own emotional responses. Specifically, these results suggest that the emotional experiences of high OC participants may be relatively “noisy,” which would make them difficult to discern clearly.

##### 4.2.2.2 Induced Monitoring

In this next study [58], we aimed to explore the effect of heightened monitoring on the experienced emotion of interpersonal closeness. We aimed to explore whether increased monitoring of an internal state would result in an attenuation of that very state (what is labeled as “try again” in Fig. (**1**) when an answer to an internal state is not clear enough). To that end, we used a well-established protocol designed to create intimacy between conversation partners by instructing them to exchange increasingly personal questions (*e.g*., “If a crystal ball could tell you the truth about yourself, your life, the future or anything else, what would you want to know?”, “When did you last cry in front of another person? By yourself?” [59]). Prior to starting the protocol, half of the participants were asked to repeatedly question and monitor their feelings of closeness to a conversation partner, while the other half were asked to monitor the room’s temperature. As predicted, repeated monitoring of the feeling of closeness appears to have diminished the level of closeness experienced by participants in this group, compared to control participants, as reflected by the physical space between partners (*i.e*., their chairs) at the end of the study.

### 4.3 Additional Internal States

#### 4.3.1 The Sense of Understanding

Feeling unsure if one understood something they read or heard is not included in the formal list of OCD symptoms. Yet, it appears to be quite common among people with this disorder and has been recognized in treatment-oriented texts on OCD (*e.g*., [60]). Here, for example, is how a person suffering from OCD describes on an OCD internet forum her experience of reading: “With each sentence, the tightness in my chest grew stronger, alongside the growing panic that “**I hadn't understood it properly or read it correctly**” [61]. What might explain this experience? In the SPIS model, this phenomenon is conceptualized as attenuated access to one’s sense of understanding.

In a recent study, we examined the prediction that OCD symptoms would be related to seeking proxies for the sense of understanding, particularly when no objective feedback on the level of understanding would be available [62]. Participants with high and low levels of OCD symptoms completed a task in which they read a fairly complicated text presented over several segments on a computer screen. While reading the text, participants were presented with a set of four proxies labeled “Learning Aids”, shown in a pilot study to be unhelpful in understanding the text^11^. In addition, only half of the participants in both the high and the low OC groups received ongoing feedback as to their level of understanding in the form of relevant comprehension questions that were followed with feedback. The other half of each group received no such feedback; We examined the number of proxies for understanding, in the form of “Learning Aids,” that participants used while reading the text. As expected, participants who did not receive feedback used more “Learning Aids” than those who received feedback during reading. More critically, and in line with the prediction of the SPIS model, high OC participants used more “Learning Aids” than did low OC participants when no ongoing feedback was available. Importantly, the two OC groups did not differ in their actual level of understanding of the text.

#### 4.3.2 Decision Making

Clinical observations suggest that OCD is associated with a marked difficulty in decision-making and higher levels of indecisiveness. In fact, decisions often constitute a major content of obsessions, which may manifest across a range of domains – from difficulty committing to a romantic partner [10] to simple perceptual decisions [63].

In the decision-making literature, two decision-making styles – “maximizing” and “satisficing” – have been contrasted, with the first marked by endlessly, and most often futilely, looking for the ever better alternative, while the latter, also considered more adaptive, involving a search for alternatives that terminates when a satisfactory result has been achieved [64, 65]. Considered from the SPIS perspective, OC symptoms should be related to the maximizing decision-making style, as feeling satisfied with the choice one has made (or with the search process one has undertaken) is a subjective internal state that would be less accessible to OC individuals.

Two studies by Oren and colleagues [66] supported this prediction. In both, participants responded to a scale that assesses the tendency to maximize within decision-making contexts (the Maximization Scale [67]) together with measures of OCD (OCI-R scores; [53]), compulsive indecisiveness [68], depression and anxiety (DASS-21; [69]. Study 1 was conducted online in a Hebrew-speaking sample, while Study 2 replicated Study 1 in an English-speaking sample. In both studies, OCD symptom levels were related to a maximizing decision-making style, even after controlling for levels of indecisiveness, depression and anxiety. Moreover, we found that reliance on external proxies in daily life (assessed by the Seeking Proxies for Internal States Inventory [70]; see below) partially accounted for this association. These results suggest that maximization is uniquely related to OCD symptom levels, beyond any associations it might have with depression and anxiety, and that it is not merely a general problem of indecisiveness. According to the SPIS model's view, OC individuals continue to search in the hope of finding an alternative that would be the best in an objective rather than a subjective way. Only this “objectively best” alternative would allow them to stop searching because it would free them from the need to access their sense of satisfaction.

#### 4.3.3 Sense of Agency (SoA)

A sense of agency (SoA) is defined as perceiving oneself as the initiator or agent of one’s actions [71]. It is based on the integration of both external and internal cues of proprioception, movement, and interoception [71-73], with weights given to different cues in this integration process depending on the relative precision of each cue [74]. As SoA depends, among other factors, on one's perception of their internal signals, the SPIS model suggests that the SoA of high OC individuals should be reduced in contexts where internal signals are the main source of information.

Several sources of evidence support this prediction. First, the studies reported above on muscle tension [6, 33, 34, 42] provide some indication for attenuation of interoceptive signals of muscle tension in both high OC and clinical OCD participants. Second, in a study specifically exploring proprioception, participants with high and low OC symptoms were required to reposition their heads to a target angle that was acquired actively (*i.e*., the participant turned his/her head until instructed to stop, generating an internal cue of proprioception) or passively (*i.e*., the experimenter turned the participant’s head to a required position). As predicted by the SPIS model, while low OC participants showed an increase in accuracy after active (compared to passive) acquisition, as their active movements presumably generated additional information, high OC participants' accuracy did not improve, presumably because the active head movement failed to produce a clear proprioceptive signal [75]. These results suggest that high OC individuals may have deficient access to the proprioceptive cues involved in active movement, which might contribute to their reduced SoA. Another study of the SoA in relation to OCD exploited *the intentional binding effect –* the finding that when an event results from one’s intentional action, it is perceived to have occurred earlier than when the same event is externally generated [76-78] – which is considered an indirect measure of SoA. In line with the SPIS model, Oren and colleagues [79] found that high OC participants showed a smaller intentional binding effect than low OC individuals [79]. The authors attributed this finding to the high OC participants' attenuated access to the internal cues mentioned above that give rise to the perception of the SoA.

### 4.4 Seeking Proxies for Internal States in Everyday Life

We also investigated how seeking proxies for internal states is expressed in day-to-day life and whether, as the SPIS model postulates, it is more common among high OC individuals and those with clinical OCD. To that end, we constructed the Seeking Proxies for Internal States Inventory (SPISI; [70]) based on clinical cases of people with OCD. The sample of internal states in the SPISI includes hunger, interpersonal closeness, preferences, and a sense of understanding. The sample of proxies includes one's behavior, the opinion of others, and objective indicators such as grades and time intervals. Some items in the SPISI refer to specific internal states and proxies (*e.g*., “To know how hungry I am, I consider what and when I’ve eaten today”; “I would prefer to use a formula to solve a math problem even if I think I know the answer”), others gage more general tendencies (*e.g*., “I look for rules that would tell me what I’m supposed to do”; “Sometimes I have to infer my feelings from my actions”).

Following its development and initial validation, the SPISI was administered, alongside measures of OCD symptoms (OCI-R) and of anxiety, depression and stress (DASS-21), to representative samples of the Israeli population ([70]; Study 1) and the Dutch population ([70]; Study 2). Results showed an identical SPISI-to-OCI-R correlation coefficient (*r* = .56) in both samples, despite the different languages. This correlation remained high in both samples after controlling for anxiety, depression and stress scores [70]. In another unpublished study, OCD participants had higher scores on the SPISI (N=28; *M* = 45.82, SD = 9.27) than non-clinical control participants (N=29; *M* = 39.27, SD = 8.81), *t*(55) = 2.73, *p* = .008, *Cohen’s d* = 0.72. These studies show that OCD is associated with difficulty in judging internal states in many real-life situations; presumably, to compensate for this difficulty, OC individuals tend to seek and rely on a myriad of external proxies for these internal states to guide their behavior.

## 5 THE SPIS MODEL – SOME OPEN QUESTIONS

The SPIS model suggests that attenuated accesses to one’s own internal states (and the accompanying obsessive doubt) drive individuals with OCD to seek and rely on proxies and that these processes conjointly lead to symptoms of OCD. While this conceptualization lies at the core of the SPIS model, additional factors may also come into play, interacting in different ways with the core factor of the SPIS model. We now briefly describe a few of these factors.

### 5.1 Possible Antecedents of Attenuated Accesses to Internal States

Both classic [80] and more recent [81, 82] theories of social-emotional development suggest that empathic sharing with and reflection of experiences by other people are critical for one’s ability to learn to access, correctly label, and understand their internal states [83-85]. Conversely, if a child typically experiences states that are not shared by others or have limited ability to share their experiences and receive empathic responses, such circumstances might disrupt the normal process of learning to access internal states. How might this occur? At least two aberrant processes may be relevant here – sensory dysregulation and impaired social communication. Sensory dysregulation is defined as the tendency to experience a wide range of stimuli as aversive. Thus, unlike normally developing children, those suffering from sensory dysregulation have inner experiences that may not be readily shared by others. As a result, learning to correctly access and label one’s sensations could be impaired, or in the SPIS model’s terminology, access to these specific internal states might become attenuated. Indeed, research has shown that sensory hypersensitivity is positively correlated with ritual frequency among children and with OCI-R scores in adults [86] and that children with atypical sensory responsiveness, compared with children with normal sensory responsiveness, demonstrate a higher frequency of ritualistic behaviors [87]. Social communication is an interactive process in which social information is exchanged and shared with others [88]. Thus, children that are not exposed to sharing and empathizing of emotions due to either deficient social skills (*e.g*., children with disorders from the autistic spectrum) or life circumstances (*e.g*., traumatic childhood environments) would likely fail to learn to access their internal states [89]. Supporting this hypothesis, OCD was found to be related to autistic spectrum disorders [5, 90-97]. Similarly, research on OCD and childhood trauma shows an association between the latter and alexithymia (*i.e*., difficulty in identifying one’s own emotions and in distinguishing between emotions and bodily sensations), with alexithymia being associated with the total number and severity of obsessions [98].

### 5.2 Other Factors that may Contribute to Attenuated Access to Internal States

As discussed earlier, the causal chain between attenuation of internal states and doubt is not a unidirectional one – not only lacking clarity about internal states may lead to doubting them, but also obsessional doubt can diminish the clarity of the very internal state that the person tries to access. Specifically, obsessive doubt may increase efforts to identify/monitor the relevant internal state, efforts which only further degrade that very experience (see Fig. (**1**); the repeated attempts to “Try again” to “access [the] internal state”). Additionally, we propose that other factors “external” to the SPIS model may play a role in the vicious cycle connecting obsessive doubt, monitoring efforts, and attenuated access to internal states.

#### 5.2.1 Cognitive Factors

Several cognitive abnormalities have been suggested to figure in the etiology and maintenance of OCD, which may also be relevant to the SPIS model. These can be roughly divided into two types [99] – dysfunctional beliefs and biases in information processing.

OCD-related Dysfunctional Beliefs may explain the heightened subjective importance assigned by the individual to specific domains of human functioning (*e.g*., cleanliness). As explained above, the SPIS model asserts that one’s potential difficulties in accessing internal states will come into play only when the OC individual faces a question about their internal states that is important for them to answer accurately. Thus, from the SPIS perspective, heightened subjective importance assigned to specific domains may lead to increased monitoring efforts of, and self-doubt in, these domains, which only serve to further diminish their clarity [6]. While this process has been exemplified in the above-reviewed studies on the effects of experimentally induced doubt and increased monitoring of one’s internal states on accessing these states [9, 32-34, 58], dysfunctional beliefs may have a similar effect by increasing the importance of specific domains. These include, among others, inflated responsibility [100] – the tendency to interpret benign intrusive thoughts [in specific domains] as indicating harm/danger and one’s responsibility to protect oneself or others; overestimation of threat [101] – assigning heightened threat or danger estimations to specific areas of functioning; over-importance of thoughts [102] – the biased belief that the mere occurrence of a thought [in a specific area of life] signals its significance; intolerance of uncertainty [102] – the distress experienced due to ambiguous (uncertain) situations and the ensuing need to resolve this ambiguity; and perfectionism – the dysfunctional belief that high standards are mandatory in certain areas [39]. Moreover, these dysfunctional beliefs may also shed light on the still open question of OCD content specificity – why is it that a general doubt in and attenuation of internal states in OCD, as suggested by the SPIS model, manifests in specific realms or worlds of content. As stated earlier, the answer may be related to the subjective importance of the relevant domain. Future research can examine how OC doubt and reliance on proxies vary with domain relevance or subjective importance [6].

Biases in information processing have also been implicated in OCD [99], with additional research showing how these biases may further degrade one’s confidence in their internal states [28, 30, 31, 103]. For example, research on attention allocation in OCD has shown OC individuals to be biased in the way they allocate their attention, favoring OCD threat-related stimuli over neutral stimuli [104]. However, research has also shown that perseverative staring may cause uncertainty about the perception of these exact stimuli [30, 31]. Similarly, research has also shown that repeated checking due to reduced confidence in one’s memory may further decrease memory confidence [28, 103]. The SPIS model would predict that this reduced confidence (in these two examples, in one’s perception or memory) may then lead to increased monitoring and hence to attenuation of the relevant internal state, culminating in a vicious cycle connecting doubt, monitoring, and attenuated access to internal states. [28, 30, 31, 103, 104].

#### 5.2.2 Social Norms

Another factor that may increase the subjective importance assigned to specific domains (*i.e*., internal states) is that of social norms. The SPIS model implies, for example, that the prevalence of Relationship-Focused Obsessive-Compulsive Disorder (ROCD) may be higher in societies that emphasize the expectation of experiencing love, attraction, and excitement toward one's intended marital partner compared to societies that prioritize objective factors over subjective feelings in the context of marriage [5]. Religion provides another example of how norms can influence the importance of particular internal states. According to the SPIS model, religions that require individuals to feel a sense of devotion or love towards a higher power or to pray with “full intent” may create fertile ground for OCD symptoms among believers who struggle with determining the adequacy of their devotion or the fullness of their intent [5]. Research has indeed demonstrated that “scrupulosity OCD” encompasses symptoms involving uncertainty about one's relevant internal state, such as questioning the genuineness of love felt towards God [105, 106]. Future research might explore these questions empirically and establish the specific contributions of social norms to the phenomenology of OCD in the view of the SPIS model.

## 6 POSSIBLE NEURAL CORRELATES OF ATTENUATED ACCESS TO INTERNAL STATES

Which brain region/s may be involved in the normal process of accessing internal states? Is there a region or network of regions in the brain that could be implicated in such diverse realms, ranging from accessing one’s bodily states to one’s emotions and feelings? One promising candidate would be the anterior insular cortex (AIC), a region located deep in the cerebral cortex [107].

Specifically, it has been suggested that interoceptive signals from within the body, initially represented at the sensory level in the posterior insula, are relayed to the anterior insula for additional processing. Within the anterior insula, the ventral anterior insula, functionally connected to limbic/paralimbic regions, plays a role in emotion processing, while the dorsal anterior insula, functionally connected to regions involved in cognitive control, is believed to be active in tasks that require effortful attention and salience detection. Thus, it has been proposed that through the integration of emotional and cognitive information with sensory information, the anterior insula carries a higher-order re-representation of interoception [108], including all subjective feelings and states that emanate from the body [109, 110]. Predictive coding/active inference theories of the mind [111] and their application to psychopathology [112] have advanced a related view, according to which the AIC is the interface between interoceptive, bottom-up information, and top-down predictions of these states from high-order cortical regions. This framework views emotion as a form of interoceptive inference; that is, subjective feelings are based on the active interpretation of changes in the physiological conditions of the body [113], which are then (at least sometimes) relegated to awareness [113]. From this perspective, the insular cortex is believed to form an interoceptive image of one’s physiological states, which is then relayed to subjective awareness of feelings [114, 115].

Empirically, the AIC has been implicated in awareness of a wide range of interoceptive states, such as thirst, hunger and pain [109, 110, 114, 116-119]. The AIC has also been specifically implicated in subjectively experiencing a wide range of emotions and feelings, such as love and happiness, disgust, risk, and uncertainty [109, 110, 120-128]. Furthermore, AIC activity was found to be associated with the saliency [129] and intensity [125] of one’s feelings and with one’s empathy for others experiencing the same emotions [128, 130-132]. Finally, deficits in emotional awareness were found to be associated with functional deficits of the AIC [133, 134].

Imaging research has also demonstrated the involvement of the insula in OCD, presumably through its numerous connections with brain regions within the cortical-striatal-thalamocortical (CSTC) pathway, which is considered to play a pivotal role in maintaining obsessions and compulsions [135-140]. Studies of structural morphometry have shown higher gray matter volumes in the anterior insula [141-144] and lower gray matter volumes in the posterior insula [144, 145] of OCD patients compared to healthy controls. Studies using a variety of functional magnetic resonance imaging (fMRI) tasks have shown OCD to be characterized by aberrant insula activity, reflected in altered connectivity and task activation (*e.g*., [146-158]. Finally, resting-state functional connectivity (rs-FC; [159, 160]) studies have shown the AIC to be less activated in OCD patients [161] and to have reduced connectivity with parts of the CSTC pathway, compared to matched healthy controls [162, 163].

Taken together, the functions of the AIC and its involvement in OCD appear consistent with the SPIS hypothesis of attenuated access to internal states in this disorder, giving rise to two classes of predictions. First, the AIC would be differentially implicated when comparing the performance of participants with high and low OC symptoms on SPIS tasks that have shown behavioral differences between these groups, for example, when completing the Experiential but not the Strategic part of the emotional intelligence test, or when asked to produce different levels of muscle tension, but only when the biofeedback monitor is not available. Second, OCD symptoms would be related to performance on tasks that have been previously shown to involve the AIC in unselected participants. For example, exploring the differences between high and low OC individuals on a heartbeat detection task (*i.e*., participants are asked to judge the timing of their heartbeats), which has shown enhanced insula activity in unselected participants, with the extent of neural activation in the right anterior insula predicting task accuracy [119]. Alas, these two lines of predictions still await formal examination in future research.

In sum, extant research on the involvement of the AIC in awareness of a wide range of internal states (*e.g*., interoceptive states, emotions and feelings) and the neurocircuitry of OCD suggests that it might play a key role in the mechanisms underlying OCD according to SPIS model. We hope that our review will encourage straightforward empirical exploration of this exciting possibility.

## 7 CLINICAL IMPLICATIONS

The SPIS model of OCD has been developed over the last decade, continuously evolving based on scientific research, as well as on clinical practice and experience, with the aim to eventually inform current psychotherapeutic practices. We believe that the SPIS model, and specifically its two main tenets (*i.e*., attenuation of internal states and seeking proxies for these states), can be fruitfully integrated with extant therapeutic approaches to OCD and used to provide patients with a novel way to conceptualize and interpret their symptoms, a way that is both functional and emphatic.

Obsessional doubt can be conceptualized in therapy as emanating from actual attenuated access to one’s own internal states rather than as indicative of real danger or a potentially looming catastrophic mistake. This conceptualization may, in turn, assist patients to more easily tolerate the doubts they experience. Indeed, better dealing with obsessional doubt is an integral part of several treatment approaches for OCD. For example, the best empirically supported form of OCD treatment, exposure and response prevention (ERP; [164-167]), requires patients to refrain from performing their rituals regardless of their doubts and ensuing fears. Another form of CBT for OCD, Inference Based Therapy, specifically targets obsessional doubt [38, 168] and has shown an association between the ability to tolerate doubt and treatment outcomes [168, 169]. Finally, the need to accept doubt is an essential component of Acceptance and Commitment Therapy (ACT; [170]), which has also been proven to be an effective treatment for OCD [171, 172].

Attenuation of internal states can also be used to explain to patients the high importance they assign to their obsessions, which in the SPIS framework are understood as emanating from diminished access to other sources of information, including emotions and motivations, that might counter these normal and common frightening thoughts [173, 174]. To illustrate, a client experiencing obsessional thoughts about smothering their crying baby could benefit from understanding that their fear surrounding these thoughts arises from their struggle to access their genuine feelings towards their child. This perspective can help the client realize that such thoughts do not necessarily indicate true or deeply rooted aggressive impulses. By adopting this viewpoint, the client may be able to inhibit attempts to neutralize or alleviate these thoughts through compulsions or “safety behaviors” [175, 176].

The SPIS model's understanding of compulsive rituals as proxies can be effectively integrated into existing cognitive-behavioral therapy (CBT) approaches for OCD. Therapists can explain to their patients how limited access to internal states can result in the adoption of compensatory behaviors, such as relying excessively on norms, rules, and rituals, and seeking external validation from others. This reframing can help patients gain a better understanding of their compulsive rituals and alleviate negative emotions and self-criticism associated with their engagement in these behaviors. Moreover, it is important to explicitly recognize the negative consequences of relying on proxies. Patients can be made aware of how this reliance can contribute to vicious cycles that further impede their access to their internal states. By acknowledging these dynamics, therapy can focus on breaking these cycles and fostering the development of healthier strategies for understanding and managing internal states.

More speculatively, specific tools and procedures may be developed to improve the ability of clients with OCD to notice and identify their internal states. One potential candidate for this goal may be mentalization-based techniques [83, 177-179], designed to improve awareness of internal experiences by integrating various mental representations of these experiences [180]. Relatedly, research has shown that mindfulness-based interventions can be efficacious in OCD (for a review and meta-analytic study, see [181]). Future studies can also examine whether resisting the habitual reliance on proxies (particularly refraining from performing behavioral or mental rituals) would eventually improve clients' perception of relevant internal states. For example, therapists can encourage clients with OCD to substitute monitoring of internal states and reliance on proxies with a more intuitive approach (*e.g*., in making decisions, acting on the first thing that comes to mind).

## CONCLUSION

In this comprehensive review paper, we presented the Seeking Proxies for Internal States (SPIS) model of OCD and its main tenets, as well as a large body of supporting experimental findings (across several domains – bodily internal states, emotions, decision-making, *etc*.) accumulated over more than a decade of research. We also described the way in which the SPIS model conceptualizes compulsive behavior and obsessional doubt, as well as additional factors that may interact with the SPIS model. While gaining much support over the years, more work is now needed to elaborate the SPIS model and its potential contributions to the field, especially with regard to two less-charted territories – clinical implications and neural underpinnings. The potential therapeutic benefits of using the SPIS model within extant approaches to OCD treatments or as the basis for novel therapeutic approaches have yet to be systematically explored, which is also true for research on the possible involvement of the AIC in tasks that tap SPIS concepts. Yet, owing to the ongoing and continuous dialogue between theoretical thinking, clinical practice, and research in developing, elaborating, and fine-tuning the SPIS model, we believe it provides solid grounds for both clinicians and researchers in the field of OCD. We truly hope that the present review of the model and its rich evidence-based foundation will encourage future research in the field.

## Figures and Tables

**Fig. (1):**
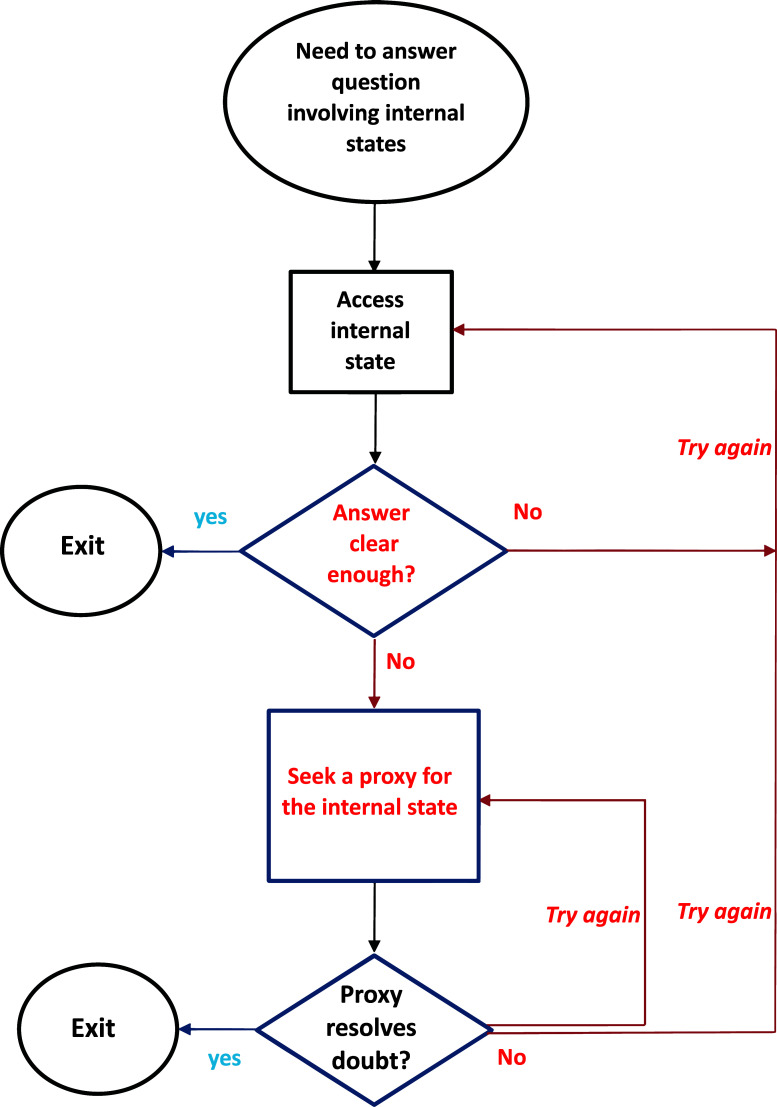
The Seeking Proxies for Internal States (SPIS) model of OCD – a schematic diagram.

## LIST OF ABBREVIATIONS

AD: Anxiety Disorders
AIC: Anterior Insular Cortex
CBT: Cognitive-behavioral Therapy
EMG: Examining Actual Physiological
GSR: Galvanic Skin Responses
NC: Non-clinical
OC: Obsessive-compulsive
OCD: Obsessive-compulsive Disorder
ROCD: Relationship-focused Obsessive-compulsive Disorder

## NOTES

1 We use “OC person” or “OC individual” to mean a person with prominent OC symptoms or with clinical OCD.

2 The SPIS model does not postulate that OC individuals are particularly prone to confront questions about internal states; rather, it suggests that when confronted with such questions, they find it more difficult to answer.

3 To quote one patient with OCD: “I don't really know that my hands are clean, but I do know that I’ve completed my washing ritual.”

4 It is important to note that no doubt would arise if access to an internal state is not compromised, as assumed to be the case for non-OC individuals, because any question regarding it would be simply resolved (see Figure 1). No doubt would arise also when there is no query, even if the person is unable to access the relevant internal state. For example, a person may have limited access to their own feelings of love, yet never ask themselves “do I love my partner?” and thus never experience doubt (per SPIS conceptualization) regarding that emotion.

5 Importantly, as will be shown, we chose tasks on which poorer performance could not be attributable solely to the presence of doubt itself.

6 Prior to the task itself, participants were trained to produce two anchor muscle tension levels – a low level, which was labeled 1 (4 microvolt) and a high level, which was labeled 4 (16 microvolt). For each of these, participants were guided to contract their forearm muscle until they have achieved the designated target. The test phase began after participants succeeded in producing both anchor levels twice. In the test phase, participants were tested on all four levels, including the intermediate levels of 2 and 3 on which they had not been trained. The interval between the training and the test phases was two minutes (except in the relaxation version, where a break was not necessary).

7 In the relaxation version GSR changes reflected on the biofeedback monitor as a moving red dot, creating a continuous line graph, with downward movement of the dot signaling an increase in relaxation, while an upward movement signaled an autonomic arousal trend indicating a reduction in relaxation. In the muscle tone version, EMG changes were visualized as the movement of a horizontal line along a vertical numerical axis ranging from 0 to 5 with intervals of 1, which corresponded to EMG values of 0 to 20 microvolts with intervals of 4).

8 The terms high and low OC participants refer to analog samples comprised of participants with high and low levels of OCD symptom, as determined by their OCI-R scores [47].

9 In all studies examining clinical samples described in the present review OCD and anxiety-disorders (AD) control participants were recruited from a community mental health center in Israel. Primary and co-morbid diagnoses were based on a formal intake interview conducted by a psychiatrist or a trained clinical psychologist using the DSM as part of the regular admission process to the mental health center. In both clinical groups, we excluded participants with present or past psychiatric diagnosis (other than anxiety disorders), specifically psychotic episodes, co-morbid post-traumatic stress disorder, tic disorder or Tourette's syndrome, neurological conditions (*e.g.*, epilepsy, brain injury), abuse of drugs or alcohol, or use of neuroleptic medication. Non-clinical control participants were assessed with the Mini-International Neuropsychiatric Interview (MINI; Sheehan DV, *et al.*, The Mini-International Neuropsychiatric Interview (MINI): The development and validation of a structured diagnostic psychiatric interview for DSM-IV and ICD-10. Journal of Clinical Psychiatry. 1998; 59: 22-33.) and were excluded for any current or past psychiatric diagnosis. All participants signed informed consent and received a small sum of money for their time.

10 For example, while items Experiential EI items may ask responders to rate which emotions are evoked by a colorful abstract picture, Strategic EI items may ask responders to indicate whether demanding more work from someone who is already stressed by their workload would produce in him/her a feeling of being “overwhelmed,” “depressed,” “ashamed,” “self-conscious” or “jittery.”

11 For example, one such "Learning Aid," labeled “Statistics,” provided the number of appearances of specific words, numbers, or linguistic structures in a certain segment of the text (*e.g.*, the number of proper nouns).
